# Knowledge map of self-reported outcomes in patients with prostate cancer: a bibliometric analysis (2014-2023)

**DOI:** 10.3389/fruro.2025.1574626

**Published:** 2025-08-15

**Authors:** Qiuxia Qin, Juan Liu, Na Zeng, Xiaoqin Xie, Fan Yang

**Affiliations:** Department of Nursing, Tongji Hospital, Tongji Medical College, Huazhong University of Science and Technology, Wuhan, Hubei, China

**Keywords:** prostate cancer, patient reported outcomes, bibliometrics, Citespace, VOSviewer, R package

## Abstract

**Objective:**

To analyze the related literature of self-reported outcomes of prostate cancer patients using bibliometric methods, and explore the research status and development trend in this field.

**Methods:**

The literature related to self-reported outcomes of prostate cancer was searched in Web of Science core database. The literature on prostate cancer self-reported outcomes was visualized using VOSviewer, CiteSpace and R software packages.

**Results:**

A total of 1119 relevant literatures were retrieved. Annual output consistently exceeded 100 articles since 2018, peaking at 161 in 2022. The U.S. (47.2%) and U.K. (21.5%) contributed 68.7% of publications. The University of Michigan emerged as the most productive institution. Collaborative networks showed strong U.S.-European ties, while Asian engagement intensified post-2020.The journal International Journal of Radiation Oncology Biology Physics (n=69) published most papers, whereas Journal of Clinical Oncology (n=48, citations=1,412) was most influential. Dual-map analysis revealed frequent citations from molecular/biology journals to clinical medicine literature. Barocas D.A., Cooperberg M.R., Koyama T., and Chen R.C. (21 publications each) were top producers. Ethan Basch (259 co-citations) was the most cited scholar. The EPIC scale development study (Wei et al., 2000) was the most co-cited reference. Key citation bursts included Taneja’s long-term outcomes study (2013-2018) and the CHHiP radiotherapy trial (2018-2021). “Quality of life” (181 occurrences) dominated keyword analysis, followed by “radiation therapy” and “prostatectomy.” Five thematic clusters emerged: radiotherapy with a blue cluster, prostatectomy with a green cluster, daily management with a red cluster, research methods with a yellow cluster and scale development with a purple cluster. Qualitative methods gained prominence after 2020, while exercise and radiotherapy remained sustained intervention focuses.

**Conclusions:**

The reported outcomes of patients with prostate cancer have continued to receive attention in the past 10 years. In this study, three recognized bibliometric software were used for the first time to analyze the related studies on the reported outcomes of patients with prostate cancer, so as to provide reference and direction for future research.

## 1 Introduction

Prostate cancer is a male genitourinary system cancer, and its incidence is the second highest among male malignant tumors (1). The incidence of prostate cancer is gradually rising. It is predicted that the number of new cases worldwide will increase from 1.4 million in 2020 to 2.9 million by 2040 (2). Actively carrying out early screening for prostate cancer, optimizing multidisciplinary collaborative diagnosis and treatment modes and whole process management can improve the diagnosis and treatment level of prostate cancer, and whole process management help improve the diagnostic and therapeutic level of prostate cancer and improve the prognosis of patients (3).

Patient reported outcomes (PROs) refers to subjective evaluations of a patient’s health status and treatment outcomes directly reported by the patient, without interpretation by healthcare professionals (4). PROs are critical indicators for assessing disease progression in cancer patients, helping healthcare providers understand patients’ health conditions and needs, promoting shared decision-making in healthcare, and improving communication between patients and providers (5). Prostate cancer patients have multiple symptoms coexisting for a long time after surgery, including urinary incontinence, sexual dysfunction, sleep disorders, anxiety, etc. as an important indicator to measure the disease outcome of prostate cancer patients, self-reported outcomes can evaluate one or more symptoms of prostate cancer patients and their impact on life from the perspective of patients (6).

With the continuous development and validation of assessment tools for self-reported outcomes of patients with prostate cancer (7, 8), alongside discussion on barriers to PRO implementation (9), and relevant statistical methods (10) have gradually gained attention. Morgans et al. highlighted the critical importance of incorporating rigorously assessed PROs for quality of life (QoL) in metastatic castration-sensitive prostate cancer (mCSPC) trials in 2019 (11). Several clinical trials and systematic reviews have meticulously supplied the impacts for different PCa treatments on urinary, sexual, and bowel function over time, providing valuable insights into the PRO profiles associated with specific treatments (12–14).

However, while these focused analyses illuminate the PRO consequences of particular interventions, key knowledge gaps remain. Existing reviews often concentrate on specific treatments or disease stages, offering a fragmented view. They largely overlook the broader landscape: How do research focus areas evolve over time? What are the prevailing intellectual structures, collaborative networks (across authors, institutions, countries), and emerging frontiers within the entire field of prostate cancer PROs? Furthermore, these analyses typically do not account for how macro-level factors like healthcare systems, national priorities, or economic contexts might influence research directions and accessibility of PRO-informed care. Crucially, they provide limited insight into the overarching knowledge structure: Where has the field been concentrated? What are the current hotspots? And most importantly, based on the entire body of literature, where should future research and clinical implementation efforts be directed to address unmet patient needs?

Bibliometric analysis offers a powerful methodological approach to address these specific gaps. As a quantitative method leveraging mathematics and statistics to analyze literature systems and their characteristics (15, 16), bibliometrics excels at mapping the intellectual structure, evolution, and collaborative dynamics of a research field.It can provide bibliometric relationships between authors, organizations, countries and references in related research fields. Common bibliometric tools include VOSviewer (17) CiteSpace (18) R Package (19). Therefore, this study uses CiteSpace, VOSviewer and R package ‘biblometrix’ to visually analyze the literature related to the reported outcomes of patients with prostate cancer, summarizes the application and development of patient reported outcomes, reveals the current development trend of patient reported outcomes in prostate cancer, explores research hotspots in this field and provides research directions for researchers of reported outcomes of prostate cancer.

## 2 Methods

### 2.1 Retrieval method

A literature search was conducted on the core collection database of Web of Science (WoSCC, https://www.webofscience.com/), covering the period from 2014 to 2023. The retrieval date is November 21, 2024.The search strategy includes two parts: one is the “prostate cancer” and the other is “patient self-reported outcomes”. The detailed search strategy is #1 AND #2. # 1 ((TS = (“prostate neoplasia” or “neoplasia, prostate” or “neoplasia, prostate” or “prostate neoplasia” or “neoplasia, prostatic” or “neoplasia, prostatic” or “neoplasia, prostatic” or “prostate cancer” or “cancer, prostate” or “cancers, prostate” or “prostate cancers” or “cancer of the prostate” or “prostatic cancer” or “cancer of the prostate” cancers, prostatic “or” prostatic cancers “or” cancer of prostate “), and #2 ((TS = (“patient reported outcome measure” or “patient reported outcomes” or “outcome, patient reported” or “patient reported outcomes” or “outcome, patient reported outcomes” or “patient reported outcomes” or “outcome, patient reported” or “patient reported outcomes” or “self management” or “management, self” or “patient reported outcomes measure” or “patient reported outcomes”). Only articles and reviews were included and the literatures with more than 10 years and non-English literatures were excluded. Repeated studies were manual verified and removed. At the same time, two authors independently evaluated the reading of all the literatures, excluded articles unrelated to the topic and concluded the controversial articles through discussion, decided by a third senior researcher.

### 2.2 Data analysis method

**Figure 1:**
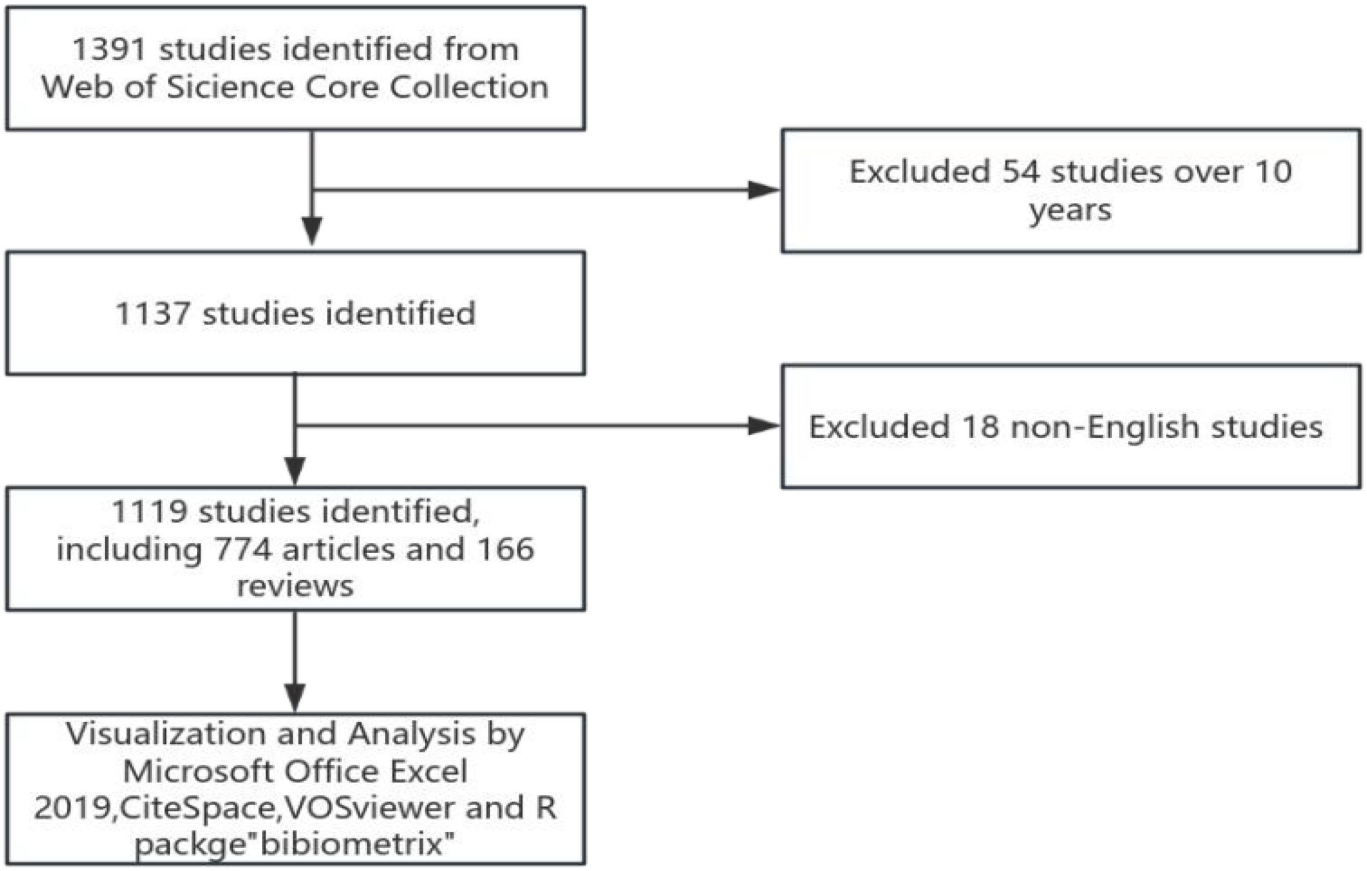
Document retrieval and analysis strategy.

VOSviewer (version 1.6.20) is a program used to construct and view bibliometric maps and to extract key information from digital publications, commonly applied to analyze author-article citation relationships, keyword co-occurrence, and other related data (20). In this paper, the software is used to analyze the relationship between authors, countries, institutions, journals, references and keywords, mapping the structural relationships. CiteSpace (version 6.2.R4) is another application that supports the use of knowledge mapping and visual exploration in bibliographic databases. In this article, CiteSpace was employed to display trends in the annual publication volume related to patient-reported outcomes in prostate cancer, create a journal co-citation overlay map, and conduct a citation burst analysis (21), revealing temporal trends. The R package “Bibliometrix” (version 4.0.0) (https://www.bibliometrix.org) facilitates thematic evolution analysis and quantitative analysis of global publication networks based on applications (22). This study utilized the Bibliometrix package for keyword burst analysis, quantifying thematic evolution. Data processing is completed by Microsoft Office Excel 2019. The specific retrieval and analysis strategies are shown in **Figure 1**.

## 3 Results

### 3.1 Document quantity analysis

**Figure 2:**
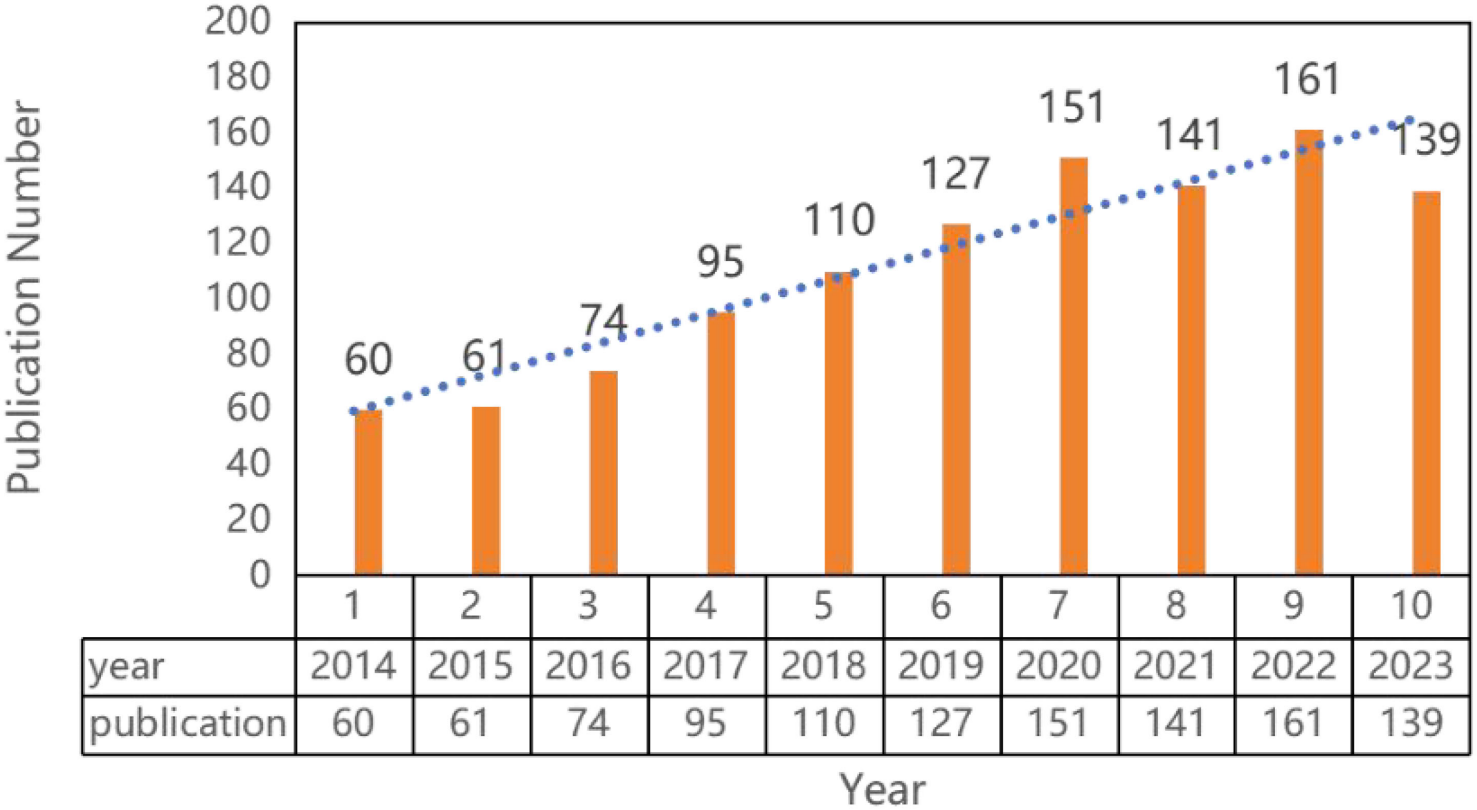
Self-reported outcomes of patients with prostate cancer in recent 10 years.

1391 literatures were screened with 54 literatures over 10 years and 18 non-English literatures excluded. Finally, 1119 literatures were included, including 774 articles and 166 reviews. Over the past 10 years, research in the field of prostate cancer outcomes has experienced an explosive growth, with the number of articles published in 2014 exceeding the total number of articles published from 1998 to 2013. Since 2018, the annual publication volume has consistently surpassed 100 articles, peaking at 161 articles in 2022, as shown in **Figure 2**.

### 3.2 National and institutional analysis

**Figure 3:**
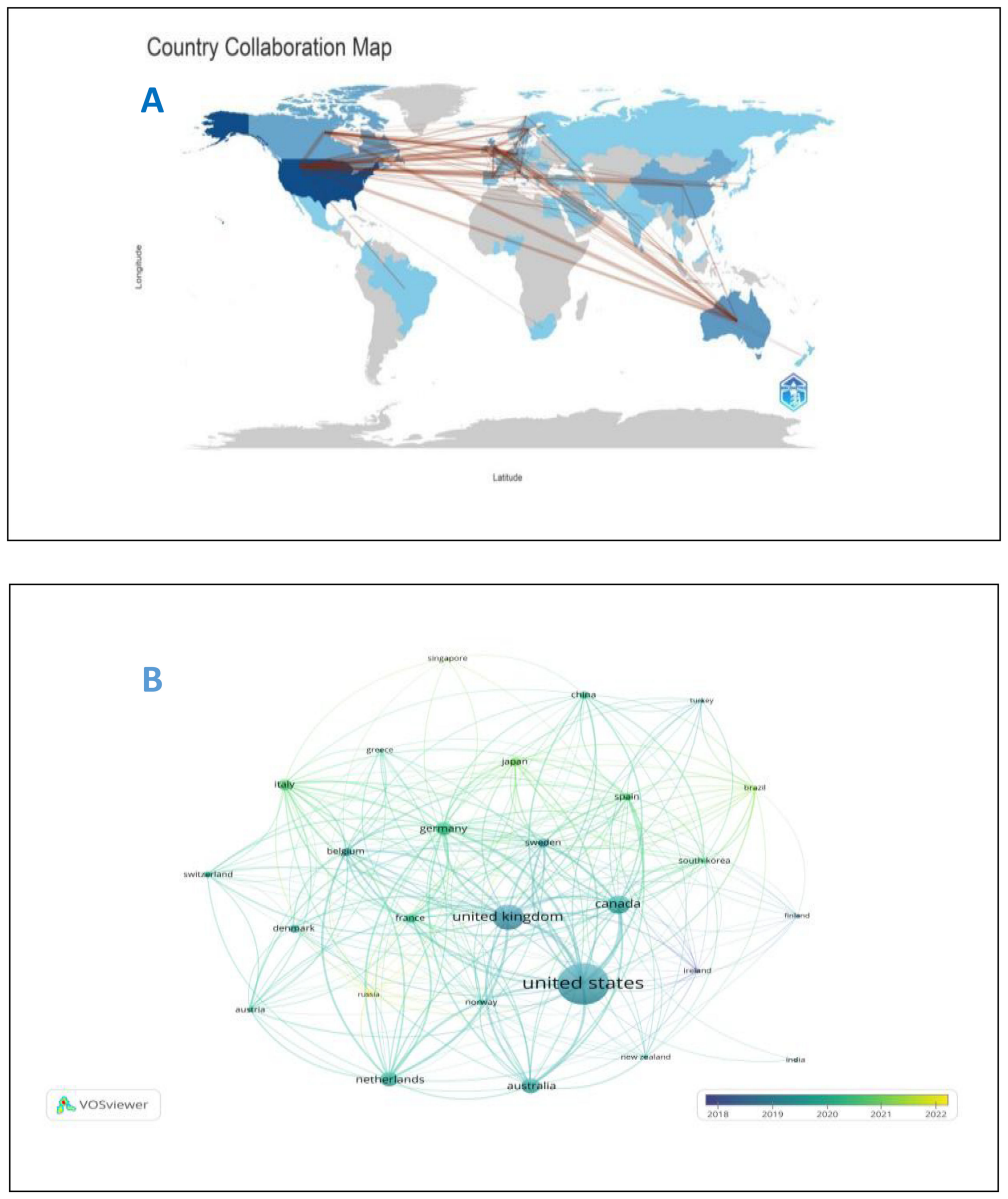
**(A)** Geographical distribution of self-reported outcomes of patients with prostate cancer. **(B)** National map of self-reported outcomes of patients with prostate cancer.

**Figure 4:**
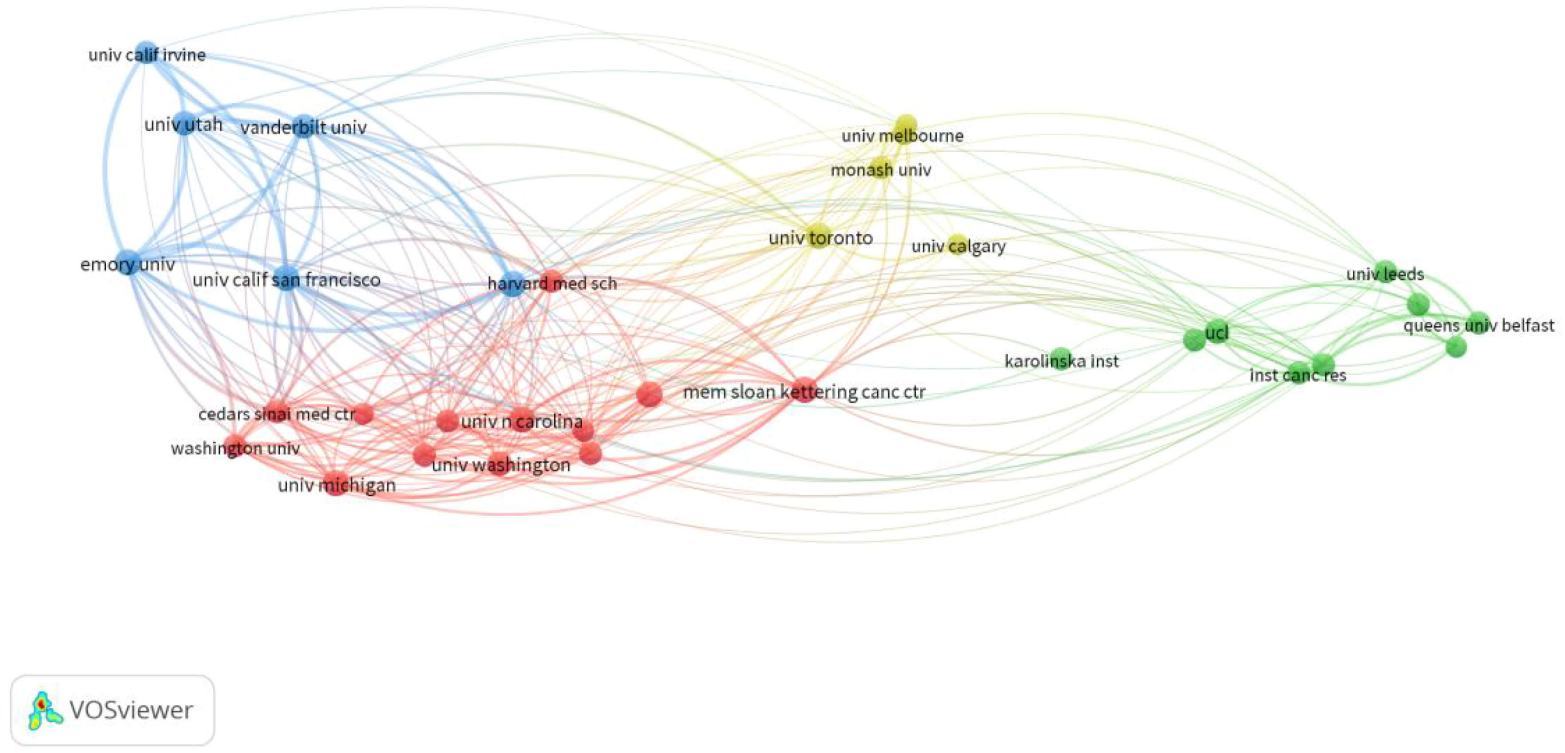
Institutional map of self-reported outcomes in patients with prostate cancer.

From 2014 to 2023, 64 countries and 2193 institutions contributed to the self-reported outcomes of patients with prostate cancer (**Table 1**) with 7 and 20 publications as thresholds, respectively.The number of papers from the United States and the United Kingdom accounts for more than half of the total number of papers (68.7%), with the United States contributing nearly half of this (47.2%). Eight of the top 10 institutions located in United States, and the other two are from Canada and the United Kingdom. **Figure 3A** shows the geographical distribution of the self-reported outcome publications among prostate cancer patients. **Figure 3B** shows a bibliometric map of collaborative relationships between 27 countries by VOSviewer. Only countries with five or more publications were included in the analysis. The United States displays close collaborations with many countries, including the United Kingdom, Canada, Australia, and Germany. Japan has closer relations with European countries and China, while India mainly collaborates with Canada and the United States, with fewer connections to other countries. Additionally, the color in the map represents the average publication year of each country’s related literature,reflecting the approximate starting time of research on self-reported outcomes in PCa patients. Finland and Ireland began related research around 2018,while the United States, the United Kingdom, Canada, and Australia entered this field and made significant progress between 2019 and 2020, leading in publication volume. Asian countries such as China, Japan, and South Korea have strengthened their research in this field between 2020 and 2021. In terms of institutional collaboration,the University of Michigan in the United States plays a significant role in this field, followed by the University of California, San Francisco. The top-ranking institution has close collaborations with several other institutions,and the University of California, San Francisco (ranked second) has strong collaborations with the University of Texas MD Anderson Cancer Center (ranked fourth). The collaborative relationships between institutions are illustrated in **Figure 4**.

**Table 1:** Top 10 countries and institutions for reporting outcomes of patients with prostate cancer.

Sort	Country	Number	Mechanism	Number
1	USA	528 (47.2%)	University of Michigan (The United States)	54 (4.8%)
2	United Kingdom	241 (21.5%)	University of California, San Francisco (The United States)	50 (4.5%)
3	Canada	149 (13.3%)	Memorial Sloan Kettering Cancer Center (The United States)	48 (4.3%)
4	Australia	108 (9.7%)	The University of Texas MD Anderson Cancer Center (The United States)	47 (4.2%)
5	Netherlands	99 (8.8%)	University of North Carolina (The United States)	46 (4.1%)
6	Germany	89 (8.0%)	Northwestern University (The United States)	45 (4.0%)
7	Italy	68 (6.1%)	University of Toronto (Canada)	45 (4.0%)
8	France	50 (4.5%)	Emory University (The United States)	43 (3.8%)
9	Japan	46 (4.1%)	University College London (The United Kingdom)	39 (3.5%)
10	Sweden	44 (3.9%)	Vanderbilt University (The United States)	35 (3.1%)

### 3.3 Journals and jointly cited journals

**Figure 5:**
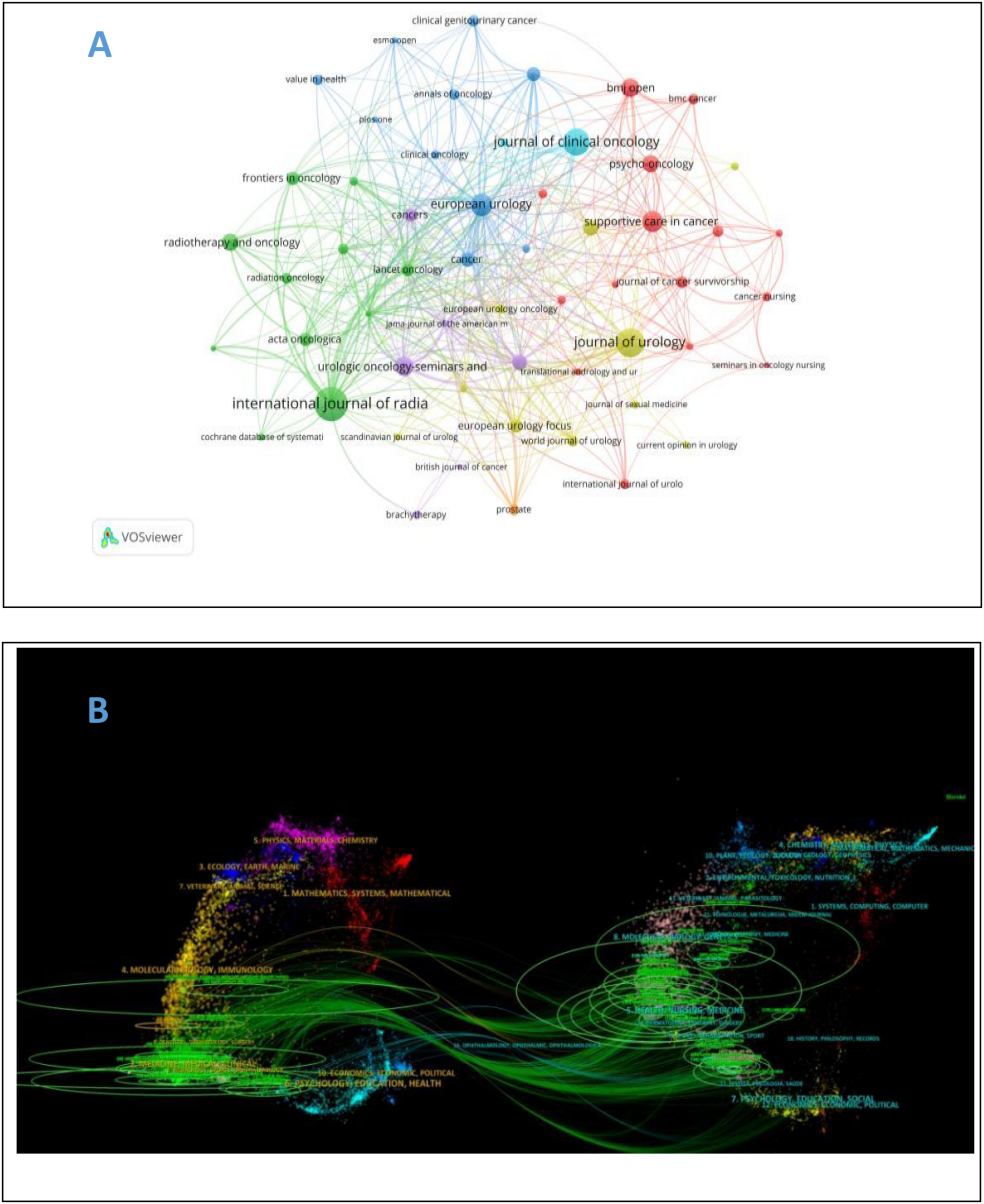
**(A)** Journal Atlas of self-reported outcomes of patients with prostate cancer. **(B)** overlay of self-reported and co cited journals of prostate cancer patients.

A total of 256 journals on self-reported outcomes of patients with prostate cancer were retrieved with more than 173 related publications. **Table 2** shows the top 10 journals with the largest number of publications and the top 10 journals with the largest number of citations. Most journals are in Q1 area. The journal with the largest number of publications is Internal Journal of Radiation Oncology Biology Physics (n = 69, IF=7.5), followed by Journal of Urology (n = 54, IF=6.8), and Journal of Clinical Oncology (n = 48, IF=44.5). Journal of Clinical Oncology ranks first in citation ranking and third in publication ranking, placing significant emphasis on self-reported outcomes in prostate cancer patients. As shown in **Figure 5A**, purple journals, such as Quality of Life Research and European Urology, have focused on prostate cancer patient-reported outcomes at an earlier stage, while yellow journals, like Prostate, have only started to pay attention to this topic in recent years. The citation relationships between journals and their co-cited journals are depicted in the CiteSpace dual-map overlay (**Figure 5B**). Each label is centered on the cluster center of the corresponding journal and represents the corresponding different principles for publishing cited articles. The left side of the figure is the collection of cited journals, and the right side is the collection of cited journals. The thickest green line indicates the strongest citation relationship in the field. These thick lines indicate that literatures published in the fields of Molecular/Biology/Genetics/Nursing/Medicine/are often cited by Molecular/Biology/Immunology/Medicine.

**Table 2:** Top 10 journals with published papers and cited top 10 journals with reported outcomes of prostate cancer patients.

Rank	Journal	Count	IF	Q	Co-cited journal	Co-citation	IF	Q
1	International Journal of Radiation Oncology Biology Physics	69	7.5	Q1	Journal of Clinical Oncology	2323	44.5	Q1
2	Journal of Urology	54	6.8	Q1	International Journal of Radiation Oncology Biology Physics	2148	7.5	Q1
3	Journal of Clinical Oncology	48	44.5	Q1	European Urology	1529	20.3	Q1
4	European Urology	36	20.3	Q1	Journal of Urology	1273	6.8	Q1
5	Supportive Care in Cancer	32	6.9	Q1	New England Journal of Medicine	1245	176.0	Q1
6	Urologic Oncology-seminars and Original Investigations	28	4.5	Q2	Lancet Oncology	922	54.4	Q1
7	BMJ open	26	2.9	Q2	Cancer	898	7.9	Q1
8	Psycho-oncology	23	5.6	Q2	Bju Intertional	812	6.5	Q1
9	Radiotherapy and Oncology	23	8.9	Q1	JAMA - Journal of the American Medical Association	741	56.3	Q1
10	Quality of Life Research	21	4.2	Q1	Psycho-oncology	651	5.6	Q1

The impact factor is the impact factor in 2024, and the journal partition refers to the JCR partition in 2024.

### 3.4 Authors and co cited authors

**Figure 6:**
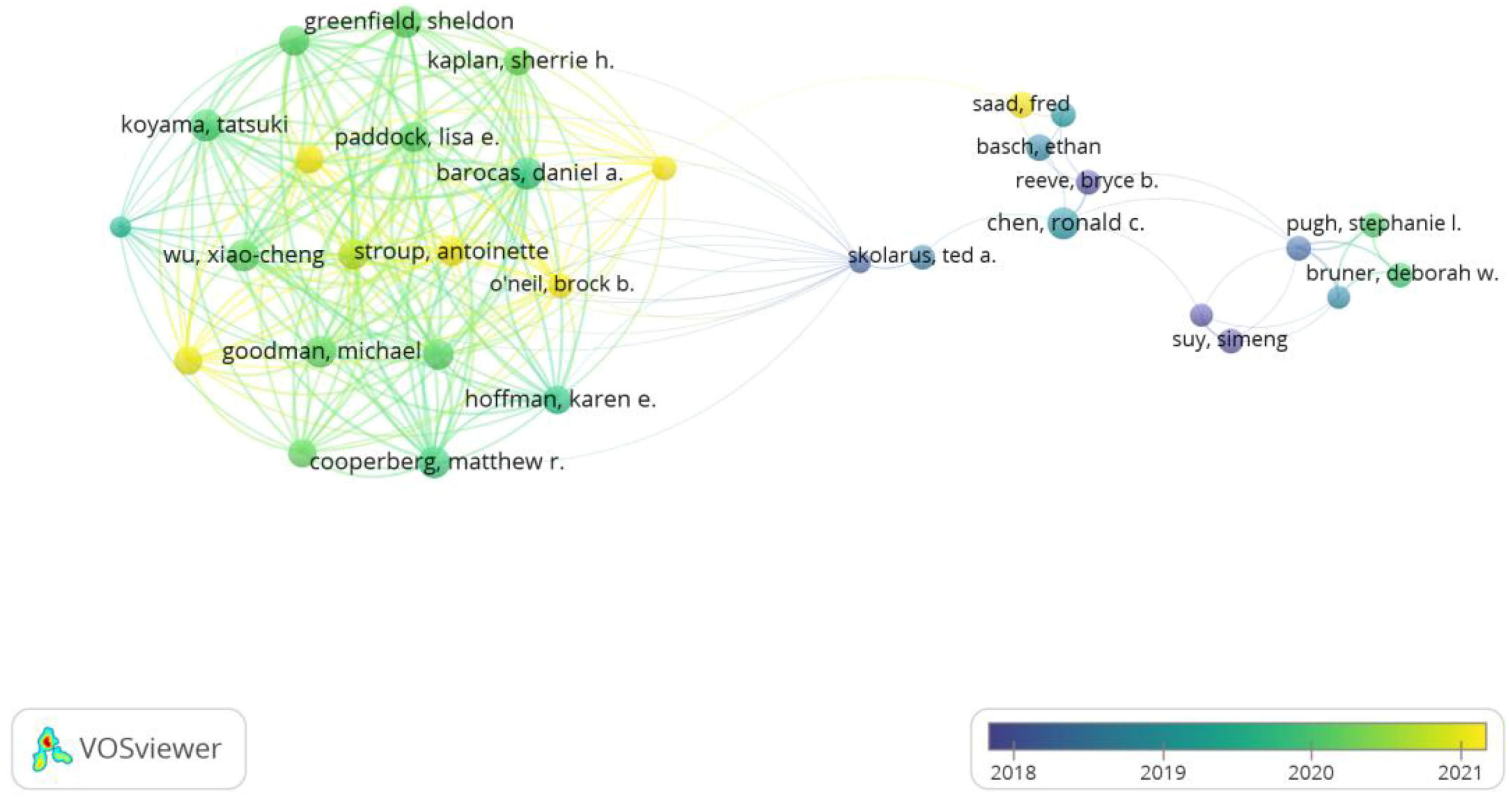
Author Atlas of self-reported outcomes of patients with prostate cancer.

6916 authors participated in the study of self-reported outcomes of patients with prostate cancer. All the top 10 authors have published at least 18 papers (**Table 3**). Barocas, Daniel a., Cooperberg, Matthew R., Koyama, Tatsuki and Chen, Ronald c. ranked first with 21 articles together.43 authors with at least 10 relevant publications were included to conduct the author bibliometrix map as shown in **Figure 6**. Barocas, Aaniel A,Cooperberg, Matthew R. and Koyama, Tatsuki showed strong cooperation with each other, while Chen, Ronald C showed plain collaboration with other experts, though he has begun related research since 2019. In addition, Ethan Basch ranks first with 259 co-citations, and the top five co-citations all exceed 140 times.

**Table 3:** Top 10 authors and cited authors of self-reported outcomes of patients with prostate cancer.

Rank	Authors	Count	Co-cited authors	Citations
1	Barocas, Daniel a	21	Basch, E	259
2	Chen, Ronald c	21	Donovan, Jl	175
3	Cooperberg, Matthew r	21	Cella, D	164
4	Koyama, Tatsuki	21	Sanda, Mg	157
5	Goodman, Michael	20	Litwin, Ms	149
6	Greenfield, Sheldon	20	Chen, Rc	144
7	Hamilton, Ann s	19	Hamdy, Fc	113
8	Paddock, Lisa e	19	Resnick, Mj	109
9	Penson, David f	19	Aaronson, Nk	106
10	Hashibe, Mia	18	Fizazi, K	105

### 3.5 Co-cited reference

Co-cited literature refers to studies that are cited together by multiple publications, reflecting the research foundation of a particular field to some extent. We selected the top 10 co-cited papers to clarify the research basis for self-reported outcomes in prostate cancer patients, as shown in **Table 4**. By constructing a co-citation map using VOSviewer, we found that among the 25,151 co-cited papers, 92 papers had been cited together more than 20 times. The most frequently co-cited paper is authored by Wei Junjie (23) from China, with their 2000 publication in Chinese Journal of Urology titled “Development and validation of the expanded prostate cancer index composite (EPIC) for comprehensive assessment of health-related quality of life in men with prostate cancer” leading the list in citation frequency. This study developed and validated a new prostate cancer quality of life assessment tool, which supplements previous measurement tools by including urinary, bowel, sexual, and hormonal symptoms, thus laying a foundation for the comprehensive assessment of self-reported outcomes in prostate cancer patients.

**Table 4:** Top 10 co-cited references on research of patient reported outcome.

Rank	Co-cited literature	Summary of article content	Citations
1	wei jt, 2000, urology, v56, p899, doi 10.1016/s0090-4295(00)00858-x	The extended prostate cancer index comprehensive assessment tool (EPIC) was developed and validated.	259
2	donovan jl, 2016, new engl j med,v375, p1425, doi 10.1056/nejmoa1606221	Objective to compare the effect of active monitoring, radical prostatectomy and hormone radiotherapy on patient reported outcomes.	209
3	sanda mg, 2008, new engl j med, v358, p1250,doi 10.1056/nejmoa074311	This study prospectively evaluated 1201 prostate cancer patients and 625 partners before and after radical prostatectomy, brachytherapy or external radiotherapy, and explored the determinants of health-related quality of life and its impact on patient and partner satisfaction.	175
4	szymanski km, 2010, urology, v76, p1245, doi 10.1016/j.urology.2010.01.027	A simplified version of the extended prostate cancer index comprehensive tool (EPIC – 26) was developed and validated to demonstrate its effectiveness and applicability.	164
5	aaronson nk, 1993, j natl cancer i, v85, p365, doi 10.1093/jnci/85.5.365	The EORTC QLQ – C30 questionnaire evaluated the quality of life of 305 patients with unresectable lung cancer in an international clinical trial. The results showed that the questionnaire had good reliability and validity.	157
6	hamdy fc, 2016, new engl j med, v375, p1415,doi 10.1056/nejmoa1606220	This study compared patients with localized prostate cancer who underwent PSA testing between 1999 and 2009. The results showed that surgery and radiotherapy had a lower incidence of disease progression and metastasis than the monitoring group.	149
7	barocas da, 2017, jama-j am med assoc, v317, p1126, doi10.1001/jama.2017.1704	The impact of radical prostatectomy, external radiotherapy, and active monitoring on patient reported functional outcomes was evaluated. It was found that after 3 years of radical prostatectomy, compared with EBRT and active monitoring, sexual function decreased more significantly, and urinary incontinence was more serious.	144
8	skolarus ta, 2015, urology, v85, p101, doi 10.1016/j.urology.2014.08.044	A score threshold of clinically relevant changes was established for the extended prostate cancer index short form (EPIC – 26), and the minimum important difference (MID) in each field of men treated for prostate cancer was analyzed.	142
9	dearnaley d, 2016, lancet oncol, v17, p1047, doi 10.1016/s1470-2045(16)30102-4	Objective to compare the efficacy and side effects of 74 Gy conventional radiotherapy with 60 Gy (20 fractions) and 57 Gy (19 fractions) high-dose fractionated radiotherapy. 60 Gy divided into 20 times is recommended as the new standard of external beam radiotherapy for local prostate cancer.	113
10	resnick mj, 2013, new engl j med, v368, p436,doi 10.1056/nejmoa1209978	Men with prostate cancer after radical prostatectomy or external radiotherapy were followed up for 2, 5, and 15 years. The results showed that surgical patients were more prone to urinary incontinence and erectile dysfunction at 2 and 5 years, but the difference was not significant at 15 years; Intestinal urgency was less common in surgical patients at 2 and 5 years, and there was no significant difference at 15 years.	111

### 3.6 Citation burst

**Figure 7:**
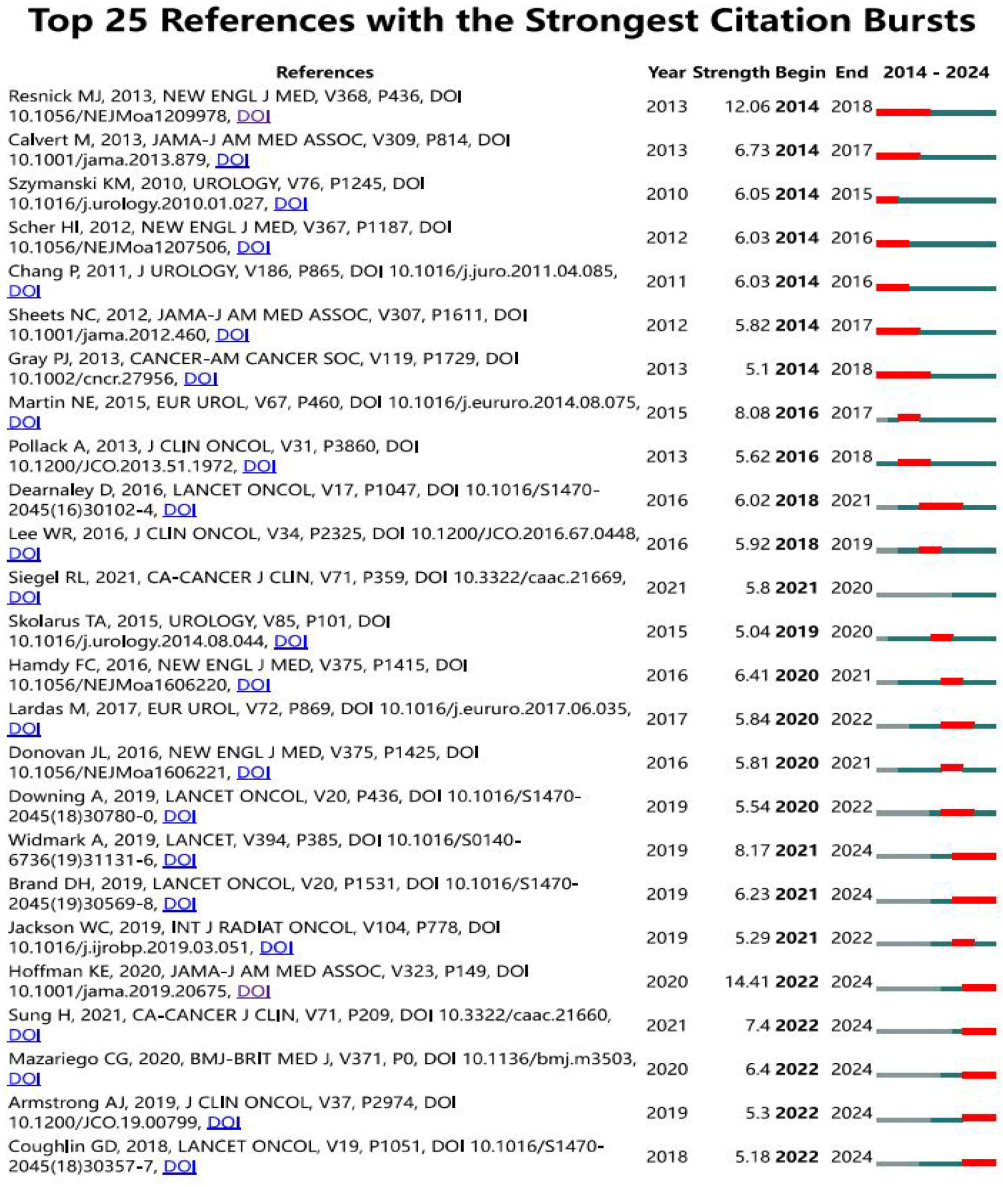
Citation explosion of prostate cancer patients’ reported outcomes.

According to the citation burst analysis in **Figure 7**, the article “Long-Term Functional Outcomes after Treatment for Localized Prostate Cancer” by Samir S. Taneja (24) published in 2013 in The New England Journal of Medicine, with high citation frequency from 2014 to 2018, and the article “Conventional versus hypofractionated high-dose intensity-modulated radiotherapy for prostate cancer (25): 5-year outcomes of the randomised, non-inferiority, phase 3 CHHiP trial” shows a log citation duration from 2018 to 2021. The authors, David Dearnaley et al. conducted the trial between October 18, 2002, and June 17, 2011, recruited 3,216 men from 71 centers. It employed a randomized controlled trial design with three groups and followed up for five years. The study utilized three different outcome measurement methods, including clinical reports and patient self-reports,to compare the proportions or cumulative incidence of side effects five years after treatment.

### 3.7 Hotspots and trends

**Figure 8:**
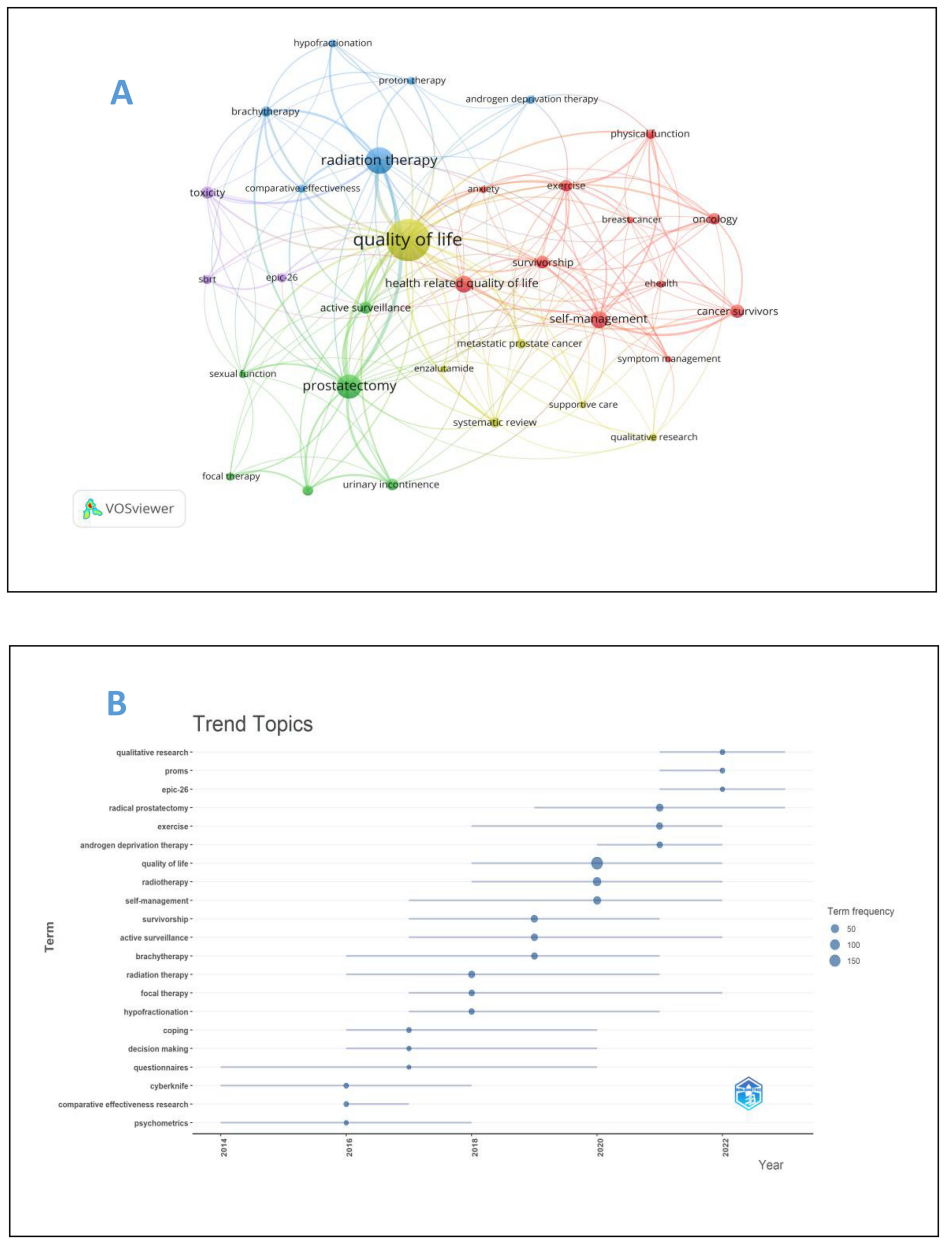
**(A)** Keyword map of prostate cancer patients’ reported outcomes. **(B)** keyword emergence map of prostate cancer patients’ reported outcomes.

The analysis of keywords is helpful to analyze the hot spots and trends in this field. A total of 1606 keywords were recorded in this study, of which 32 keywords appeared at least 13 times, and the five most frequently used keywords were “quality of life” “radiation therapy” “prostatectomy” “self-management” “health related quality of life”, **Table 5** presents the top 20 keywords. **Figure 8A** for details. As can be seen from **Figure 8A**, the word “quality of life” takes the leading role in the keyword occurrence bibliometric map. In addition, we found that the research on self-reported outcomes of patients with prostate can be roughly divided into five different fields, which are presented in different colors in **Figure 8A**, namely radiotherapy related fields (blue), prostatectomy related fields (green), daily management fields (red), research methods fields (yellow), and scale research related fields (purple). Emergent words refer to keywords that have been paid a lot of attention in a certain period of time. **Figure 8B** reflects emergent words and their duration. It can be seen from **Figure 8B** that questionnaire survey is the main research method in the early stage of prostate patients’ reported outcomes, and qualitative research has also become a research hotspot after 2020. Exercise, radiation therapy, and radiation therapy were the main intervention methods of concern from 2018 to 2022, and the research in the field of reported outcomes of patients with prostate cancer lasted for a long time.

**Table 5:** Top 20 high-frequency keywords of self-reported outcomes of patients with prostate cancer.

Rank	Keywords	Counts	Total linkstrength
1	quality of life	181	169
2	radiation therapy	84	109
3	prostatectomy	75	97
4	self-management	43	51
5	health related quality of life	43	23
6	cancer survivors	29	37
7	active surveillance	25	38
8	toxicity	25	29
9	exercise	23	37
10	urinary incontinence	23	30
11	brachytherapy	20	39
12	erectile dysfunction	20	33
13	systematic review	20	24
14	androgen deprivation therapy	15	8
15	physical activity	14	22
16	proton therapy	14	15
17	focal therapy	14	12
18	hypofractionation	13	17
19	anxiety	12	17
20	ehealth	12	17

## 4 Discussion

### 4.1 The research on the reported outcomes of patients with prostate cancer in the past 10 years is hot

The self-reported outcome of prostate cancer patients can evaluate the curative effect from the perspective of patients. It is the basis of the patient-centered diagnosis and treatment model. Historically, PROs were underutilized in clinical decisions. A national survey revealed that only 25% of urologists and radiation oncologists routinely assessed health-related quality of life (HRQOL) for prostate cancer patients in practice, partly due to challenges in integrating PROs into clinical workflows (26). However, growing recognition of PROs’ value has spurred institutional adoption. Initiatives like the Europa Uomo Patient Reported Outcome Study (EUPROMS 2.0) now systematically collect patient-reported physical/mental well-being data to support shared decision-making—demonstrating how healthcare systems increasingly prioritize patient voices (27). This shift is reflected in sustained scholarly engagement over the past decade. The increasing trend in the number of publications, especially the exponential growth in the past five years, highlights the recognition that PROs are important indicators for evaluating treatment efficacy from a patient perspective and advancing precision oncology. While 2023 saw a slight decline in publications compared to peak years (2021-2022), this fluctuation does not diminish the field’s vitality. On the contrary, it signals a natural consolidation stage following rapid expansion. The persistent high research activity confirms PROs as an enduring priority in prostate cancer outcomes assessment, necessitating continued efforts to map knowledge trajectories and emerging frontiers.

### 4.2 Main characteristics and contents of research in related fields of prostate patients’ reported outcomes

From the perspective of national and institutional publications, the United States and the United Kingdom are at the forefront of research on outcome reporting in prostate cancer patients, which is largely due to their early establishment of regulatory frameworks and standardized assessment protocols. As early as 2006, The U.S. Food and Drug Administration (FDA) officially released the “Draft Guidance on the Application of PROs in New Drug Development and Efficacy Evaluation” (28), which clearly stipulates that patients’ self-reported health outcome indicators are necessary indicators in clinical efficacy evaluation and drug trial reports. The National Health Service (NHS) in the UK uses patient self-reported data to assess the service quality of medical institutions (29). In addition, the two sides also actively developed evaluation scales more suitable for their own countries. For example, Barry DeWitt (30) established a social preference scoring system covering seven areas (cognitive function, depression, fatigue, pain interference, physical function, sleep disorders and social role participation ability) based on the preferences of the American population in 2018.In 2022, John brazier (31) conducted qualitative interviews with 168 patients and social security users in six countries, combined with psychometric testing and stakeholder consultation, and finally determined 25 items and 9 short version items, and proposed a health and well-being measurement tool. Such standardization enabled rigorous, comparable PRO data collection, directly facilitating high-impact clinical research. In contrast, China only proposed the inclusion of PROs as one of the top ten common sources of real-world data in 2020 and 2021, which corroborates the conclusion from **Figure 3B** that China began intensifying research in this area between 2020 and 2021.

Judging from the publication and citation of journals in this field, the top ten journals in both are Q1 or Q2 journals, indicating that the research quality in this field is excellent. Meanwhile, the highly cited literatures are also clinical trial articles, indicating that the conclusions of articles in this field are true and reliable, but it also means that PRO studies require longitudinal designs and multidisciplinary coordination, limiting feasibility for resource-constrained settings. It is worth mentioning that EPIC (32), EPIC-26 (33) and EORTC QLQ-C30 (34) mentioned in **Table 4** are currently the most authoritative comprehensive evaluation tools for prostate cancer index in the world. It is worth noting that due to the large number of items and the long processing time, EPIC has certain limitations in clinical application. Moreover, EPIC-26 does not take into account the psychological state of patients, while EORTC QLQ-C30, as a universal tool for assessing the quality of life of cancer patients, lacks specificity. The other seven articles are all about the evaluation of clinical patient reported outcomes of patients with prostate cancer resection and/or radiotherapy, which highlights that radical prostatectomy and radiotherapy remain the primary treatments in prostate cancer outcome research. For example, Resnick MJ (35) published a study in the New England Journal of medicine in 2013, comparing the differences in long-term urinary incontinence, intestinal function and sexual function between radical prostatectomy and radiotherapy patients. However, this focus risks overlooking novel modalities such as focal therapy or underserved populations. Different from the traditional doctor’s perspective to evaluate the impact of these two treatment methods on the survival rate of patients, the patient-centered concept of using patient reported outcomes as the evaluation results is helpful to improve the quality of life of patients with medical treatment.

### 4.3 Research hotspots and trends in related fields of prostate cancer patient reported outcomes

Our bibliometric analysis reveals three major trends in current prostate cancer PRO research. First, interventions are becoming more personalized. While surgery (e.g., “prostatectomy”) and radiation therapy remain central, interest in exercise (36), active surveillance (37), and proton therapy (38) has grown significantly. This transformation stems from advancements in diagnosis and treatment, changing treatment goals from “survival rates” to “quality of life.” It reflects a transition toward patient-centered care, with research now focusing on reducing side effects including urinary incontinence and erectile dysfunction, and developing less invasive approaches to improve long-term patient experiences. Second, outcome assessment is expanding into multiple dimensions. Unlike early studies limited to toxicity or physical symptoms, current PRO research integrates genomic risk analysis (39), dietary interventions (40), eHealth, and mental health metrics (41). This multidimensional approach evaluates patients’ well-being from both physical and psychological perspectives, highlighting PRO’s role in cancer survivorship care. Future studies may develop additional indicators to fully capture patient life experiences. Third, research methods are continuously innovating. Since 2020, qualitative research has emerged alongside traditional quantitative methods. Applications of artificial intelligence (AI) and telemedicine (42, 43) improve data collection efficiency and scope. These technologies help doctors access patient feedback more easily while strengthening patients’ active role in their care, promoting a “collaborative doctor-patient” model. Notably, prostate cancer PRO keywords show strong connections to bladder cancer (44), breast cancer (45), and rectal cancer (46). This overlap arises from: shared urinary system comorbidities (bladder cancer), breast development risks after androgen deprivation therapy (47), increased colorectal cancer incidence post-radiation, and common postoperative bowel symptom management needs. This raises a critical question: Should future prostate cancer PRO tools integrate quality-of-life dimensions from other cancers? Should we conduct a quality of life assessment based on the prediction of symptoms, in order to select a more appropriate assessment tool?

However, three key challenges persist. First, Lots of patient self-report outcome assessment tools only concentrate on assessing patients’ symptoms. They don’t pay enough attention to evaluating their mental state and social function. The widely used scales such as EPIC-26, prioritize physical symptoms while neglecting psychosocial and cultural dimensions. This narrow focus is compounded by methodological fragmentation: translating legacy instruments like anxiety scales into unified systems like PROMIS remains challenging, as evidenced by efforts to map MAX-PC onto PROMIS metrics for clinical comparability (48). However, the more comprehensive the focus dimension is, the longer it takes to conduct the scale assessment. How to balance the assessment time and the comprehensiveness of the assessment is a huge challenge. To address this, three strategies emerge from recent research: (i) enhance accuracy through decision aids, particularly for patients with low e-health literacy. A study demonstrated that decision aids reduced sexual dysfunction botherness post-prostatectomy (49); (ii) Integrate socioeconomic context into PRO frameworks. Pretreatment functional status (sr-FS) varies significantly between patients and providers, necessitating collection of social determinants for equitable care (50); and (iii) Develop next-generation tools like the PRIME framework, which uses machine learning to analyze online patient discussions for multidimensional QoL assessment (51).

Second, PRO research concentration in high-income regions obscures critical disparities including the racial differences and economic determinants. For example, black patients exhibit superior radiation sensitivity versus White patients in equal-access setting**s** (52). Besides, patients from deprived neighborhoods report lower baseline QoL, poorer health trajectories, and reduced survival (53). These findings underscore that current PRO tools fail to capture contextual barriers such as financial toxicity and rural healthcare access). PROMIS enables population-level symptom benchmarking (54), but global equity requires validation in resource-limited settings.

Thirdly, despite AI/telemedicine potential (55, 56), real-world integration lags, which may be improved based on ePRO systems for remote monitoring that protect privacy while personalizing care (57), multimodal validation combining subjective PROs with objective measures (58), and novel therapy evaluation using EMR-integrated PROs to compare next-generation ARIs like darolutamide (59).

## 5 Conclusion

Over the past decade, research on PROs in PCa has solidified as a critical field, primarily driven by the need to understand the quality of life impacts of diverse treatments. Key findings reveal a sustained focus on evaluating specific burdens: radical prostatectomy is strongly associated with sexual dysfunction and urinary incontinence, while radiotherapy often leads to bowel complications, and hormone therapy presents systemic challenges like fatigue. Current research emphasizes comparing these treatment-specific PRO profiles, particularly weighing the benefits and drawbacks of interventions like surgery or radiation against active surveillance. Methodologically, the field shows convergence, integrating longitudinal studies with cross-sectional data and increasingly utilizing mixed-methods approaches to refine PRO assessment tools, and effectively integrate the self-report outcome assessment with clinical treatment. Emerging trends point towards the development and validation of PRO measures for novel therapies such as precision surgery and proton therapy, exploring the long-term effects of these advanced interventions, and investigating individualized symptom management strategies, including digital health applications. This bibliometric analysis provides the first comprehensive mapping of this evolving landscape. It underscores the centrality of treatment-specific quality-of-life assessment and highlights shifting priorities towards personalized and technologically supported PRO research. For future work, efforts should prioritize validating PRO tools for newer treatments, optimizing digital platforms for real-world symptom monitoring and patient engagement, and rigorously evaluating the implementation of PRO findings into tailored survivorship care pathways to directly improve patient well-being.

## 6 Limitations

This bibliometric analysis may have a few limitations. Firstly, the data of this study are only from WoSCC Database, unable to completely overwrite relevant publications. Secondly, this analysis employed broad inclusion criteria to capture the full PRO landscape, requiring only that studies written in English were focused on prostate cancer, explicitly measured PROs and peer-reviewed publications, which help reduce the risk of omit seminal PRO validation papers. However, the broad inclusion criteria actually may dilute the visibility of high-impact, practice-changing studies. Notably, this analysis method is not feasible to analyze the country-specific research focus but supply an overall research focus, which may ignore the economics and culture influence on research trends in different countries. Our analysis also did not systematically categorize studies by cancer stage, which may obscure distinct PRO priorities across disease spectra.

## Data Availability

The original contributions presented in the study are included in the article/supplementary material. Further inquiries can be directed to the corresponding author.

## References

[1] Mottet N van den Bergh RCN Briers E Van den Broeck T Cumberbatch MG De Santis. EAU-EANM-ESTRO-ESUR-SIOG Guidelines on Prostate Cancer-2020 Update. Part 1: Screening, Diagnosis, and Local Treatment with Curative Intent. Eur Urol. (2021) 79:243–62. doi: 10.1016/j.eururo.2020.09.042, PMID: 33172724

[2] James ND Tannock I N'Dow J Feng F Gillessen S Ali. The Lancet Commission on prostate cancer: planning for the surge in cases. Lancet. (2024) 403:1683–722. doi: 10.1016/S0140-6736(24)00651-2, PMID: 38583453 PMC7617369

[3] Cornford P van den Bergh RCN Briers E Van den Broeck T Brunckhorst O Darraugh. EAU-EANM-ESTRO-ESUR-ISUP-SIOG Guidelines on Prostate Cancer-2024 Update. Part I: Screening, Diagnosis, and Local Treatment with Curative Intent. Eur Urol. (2024) 86:148–63. doi: 10.1016/j.eururo.2024.03.027, PMID: 38614820

[4] Guidance for industry: patient-reported outcome measures: use in medical product development to support labeling claims: draft guidance. Health Qual Life Outcomes. (2006) 4:79. doi: 10.1186/1477-7525-4-79, PMID: 17034633 PMC1629006

[5] Lv J Wang A. Application of machine learning in self-reported outcomes of cancer patients. Chin J Nurs Educ. (2019) 21:1018–24. doi: 10.3761/j.issn.1672-9234.2024.08.020

[6] Msaouel P Gralla RJ Jones RA Hollen PJ. Key issues affecting quality of life and patient-reported outcomes in prostate cancer: an analysis conducted in 2128 patients with initial psychometric assessment of the prostate cancer symptom scale (PCSS). BMJ Support Palliat Care. (2017) 7:308–15. doi: 10.1136/bmjspcare-2016-001146, PMID: 28167656 PMC5545159

[7] Murphy KM Sauer C Yang D Hass N Novakovic K Helfand. The Development of iManage-PC, an Online Symptom Monitoring and Self-management Tool for Men With Clinically Localized Prostate Cancer. Cancer Nurs. (2022) 45:E309–19. doi: 10.1097/NCC.0000000000000948, PMID: 33867430 PMC8497651

[8] Shao K Hongxia GE Shi L. Research progress of self-reported outcome assessment tools for patients with prostate cancer. J Advanced Nurs. (2019) 39:1097–102. doi: 10.16821/j.cnki.hsjx.2024.10.016

[9] Nguyen H Butow P Dhillon H Sundaresan P. A review of the barriers to using Patient-Reported Outcomes (PROs) and Patient-Reported Outcome Measures (PROMs) in routine cancer care. J Med Radiat Sci. (2021) 68:186–95. doi: 10.1002/jmrs.421, PMID: 32815314 PMC8168064

[10] Qian Y Walters SJ Jacques R Flight L. Comprehensive review of statistical methods for analysing patient-reported outcomes (PROs) used as primary outcomes in randomised controlled trials (RCTs) published by the UK's Health Technology Assessment (HTA) journal (1997-2020). BMJ Open. (2021) 11:e051673. doi: 10.1136/bmjopen-2021-051673, PMID: 34489292 PMC8422492

[11] Morgans AK Stockler MR. Patient-reported Outcomes in Metastatic Castration-sensitive Prostate Cancer in the Adjuvant Setting. Eur Urol Focus. (2019) 5:144–6. doi: 10.1016/j.euf.2018.12.007, PMID: 30612936 PMC6658671

[12] Ávila M Patel L López S Cortés-Sanabria L Garin O Pont À. Patient-reported outcomes after treatment for clinically localized prostate cancer: A systematic review and meta-analysis. Cancer Treat Rev. (2018) 66:23–44. doi: 10.1016/j.ctrv.2018.03.005, PMID: 29673922

[13] Westerhoff JM Lalmahomed TA Meijers LTC Henke L Teunissen FR Bruynzeel AME. Patient-Reported Outcomes Following Magnetic Resonance-Guided Radiation Therapy for Prostate Cancer: A Systematic Review and Meta-Analysis. Int J Radiat Oncol Biol Phys. (2024) 120:38–48. doi: 10.1056/NEJMoa1606221, PMID: 38838994

[14] Donovan JL Hamdy FC Lane JA Mason M Metcalfe C Walsh. Patient-Reported Outcomes after Monitoring, Surgery, or Radiotherapy for Prostate Cancer. N Engl J Med. (2016) 375:1425–37. doi: 10.1016/j.ijrobp.2024.05.028, PMID: 27626365 PMC5134995

[15] Zeng N Sun JX Liu CQ Xu JZ An Y Xu MY. Knowledge mapping of application of image-guided surgery in prostate cancer: a bibliometric analysis (2013-2023). Int J Surg. (2024) 110:2992–3007. doi: 10.1097/JS9.0000000000001232, PMID: 38445538 PMC11093506

[16] Li Y-M Li X-T Yu Q. A large sample bibliometric analysis of nurses' health cohort study. Chin J Nurs. (2019) 59:330–7. doi: 10.3761/j.issn.0254-1769.2024.03.012

[17] Li D Yu D Li Y Yang R. A bibliometric analysis of PROTAC from 2001 to 2021. Eur J Med Chem. (2022) 244:114838. doi: 10.1016/j.ejmech.2022.114838, PMID: 36274273

[18] Ding H Wu C Liao N Zhan Q Sun W Huang Y. Radiomics in Oncology: A 10-Year Bibliometric Analysis. Front Oncol. (2021) 11:689802. doi: 10.3389/fonc.2021.689802, PMID: 34616671 PMC8488302

[19] Yang K Hu Y Qi H. Digital Health Literacy: Bibliometric Analysis. J Med Internet Res. (2022) 24:e35816. doi: 10.2196/35816, PMID: 35793141 PMC9301558

[20] Huang Y Liao C Shen Z Zou Y Xie W Gan Q. A bibliometric insight into neoadjuvant chemotherapy in bladder cancer: trends, collaborations, and future avenues. Front Immunol. (2024) 15:1297542. doi: 10.3389/fimmu.2024.1297542, PMID: 38444854 PMC10912866

[21] Liu X Zhao S Tan L Tan Y Wang Y Ye Z. Frontier and hot topics in electrochemiluminescence sensing technology based on CiteSpace bibliometric analysis. Biosens Bioelectron. (2022) 201:113932. doi: 10.1016/j.bios.2021.113932, PMID: 35065388

[22] Zhu Z Zhou Y Li H Xu W Wang T Liu J. Research trends and hotspots in prostate cancer associated exosome: a bibliometric analysis. Front Oncol. (2023) 13:1270104. doi: 10.3389/fonc.2023.1270104, PMID: 38090502 PMC10712200

[23] Wei JT Dunn RL Litwin MS Sandler HM Sanda MG. Development and validation of the expanded prostate cancer index composite (EPIC) for comprehensive assessment of health-related quality of life in men with prostate cancer. Urology. (2000) 56:899–905. doi: 10.1016/s0090-4295(00)00858-x, PMID: 11113727

[24] Taneja SS. Re: Long-term functional outcomes after treatment for localized prostate cancer. J Urol. (2013) 190:1764–5. doi: 10.1016/j.juro.2013.07.084, PMID: 24120779

[25] Dearnaley D Syndikus I Mossop H Khoo V Birtle A Bloomfield D. Conventional versus hypofractionated high-dose intensity-modulated radiotherapy for prostate cancer: 5-year outcomes of the randomised, non-inferiority, phase 3 CHHiP trial. Lancet Oncol. (2016) 17:1047–60. doi: 10.1016/S1470-2045(16)30102-4, PMID: 27339115 PMC4961874

[26] Hamoen EHJ De Rooij M Witjes JA Barentsz JO Rovers MM. Measuring health-related quality of life in men with prostate cancer: A systematic review of the most used questionnaires and their validity. Urol Oncol. (2015) 33:19–69. doi: 10.1016/j.urolonc.2013.10.005, PMID: 24433753

[27] Hamoen EHJ De Rooij M Witjes JA Barentsz JO Rovers MM. The Europa Uomo Patient Reported Outcome Study 2.0-Prostate Cancer Patient-reported Outcomes to Support Treatment Decision-making. Eur Urol Focus. (2023) 9:1024–36. doi: 10.1016/j.euf.2023.05.006, PMID: 37268512

[28] Guidance for industry: patient-reported outcome measures: use in medical product development to support labeling claims: draft guidance. Health Qual Life Outcomes. (2006) 4:79. doi: 10.1186/1477-7525-4-79, PMID: 17034633 PMC1629006

[29] Holch P Warrington L Bamforth LCA Keding A Ziegler LE Absolom K. Development of an integrated electronic platform for patient self-report and management of adverse events during cancer treatment. Ann Oncol. (2017) 28:2305–11. doi: 10.1093/annonc/mdx317, PMID: 28911065 PMC5834137

[30] Dewitt B Feeny D Fischhoff B Cella D Hays RD Hess R. Estimation of a Preference-Based Summary Score for the Patient-Reported Outcomes Measurement Information System: The PROMIS((R))-Preference (PROPr) Scoring System. Med Decis Making. (2018) 38:683–98. doi: 10.1177/0272989X18776637, PMID: 29944456 PMC6502464

[31] Brazier J Peasgood T Mukuria C Marten O Kreimeier S Luo. The EQ-HWB: Overview of the Development of a Measure of Health and Wellbeing and Key Results. Value Health. (2022) 25:482–91. doi: 10.1016/j.jval.2022.01.009, PMID: 35277337

[32] Wei JT Dunn RL Litwin MS. Development and validation of the expanded prostate cancer index composite (EPIC) for comprehensive assessment of health-related quality of life in men with prostate cancer. Urology. (2000) 56:899–905. doi: 10.1016/s0090-4295(00)00858-x, PMID: 11113727

[33] Skolarus TA Dunn RL Sanda MG Chang P Greenfield TK Litwin MS. Minimally important difference for the Expanded Prostate Cancer Index Composite Short Form. Urology. (2015) 85:101–5. doi: 10.1016/j.urology.2014.08.044, PMID: 25530370 PMC4274392

[34] Aaronson NK Ahmedzai S Bergman B Bullinger M Cull A Duez. The European Organization for Research and Treatment of Cancer QLQ-C30: a quality-of-life instrument for use in international clinical trials in oncology. J Natl Cancer Inst. (1993) 85:365–76. doi: 10.1093/jnci/85.5.365, PMID: 8433390

[35] Resnick MJ Koyama T Fan KH Albertsen PC Goodman M Hamilton AS. Long-term functional outcomes after treatment for localized prostate cancer. N Engl J Med. (2013) 368:436–45. doi: 10.1056/NEJMoa1209978, PMID: 23363497 PMC3742365

[36] Houben LHP Overkamp M Van Kraaij P Trommelen J Van Roermund JGH DE Vries P. Resistance Exercise Training Increases Muscle Mass and Strength in Prostate Cancer Patients on Androgen Deprivation Therapy. Med Sci Sports Exerc. (2023) 55:614–24. doi: 10.1249/MSS.0000000000003095, PMID: 36534950 PMC9997646

[37] Zaorsky NG Allenby T Lin J Rosenberg J Simone NL Schmitz KH. Exercise Therapy and Radiation Therapy for Cancer: A Systematic Review. Int J Radiat Oncol Biol Phys. (2021) 110:973–83. doi: 10.1016/j.ijrobp.2020.11.024, PMID: 33220396 PMC13391086

[38] Berthold J Pietsch J Piplack N Khamfongkhruea C Thiele J Hölscher T. Detectability of Anatomical Changes With Prompt-Gamma Imaging: First Systematic Evaluation of Clinical Application During Prostate-Cancer Proton Therapy. Int J Radiat Oncol Biol Phys. (2023) 117:718–29. doi: 10.1016/j.ijrobp.2023.05.002, PMID: 37160193

[39] Berthold J Pietsch J Piplack N Khamfongkhruea C Thiele J Hölscher T. Validation of a 22-Gene Genomic Classifier in Patients With Recurrent Prostate Cancer: An Ancillary Study of the NRG/RTOG 9601 Randomized Clinical Trial. JAMA Oncol. (2021) 7:544–52. doi: 10.1001/jamaoncol.2020.7671, PMID: 33570548 PMC7879385

[40] Su ZT Mamawala M Landis PK de la Calle CM Shivappa N Wirth M. Diet Quality, Dietary Inflammatory Potential, and Risk of Prostate Cancer Grade Reclassification. JAMA Oncol. (2024) 10:1702–6. doi: 10.1001/jamaoncol.2024.4406, PMID: 39418052 PMC11581523

[41] James C Brunckhorst O Eymech O Stewart R Dasgupta P Ahmed. Fear of cancer recurrence and PSA anxiety in patients with prostate cancer: a systematic review. Support Care Cancer. (2022) 30:5577–89. doi: 10.1007/s00520-022-06876-z, PMID: 35106656 PMC9135793

[42] van Oosterom MN Meershoek P KleinJan GH Hendricksen K Navab N van de Velde. Navigation of Fluorescence Cameras during Soft Tissue Surgery-Is it Possible to Use a Single Navigation Setup for Various Open and Laparoscopic Urological Surgery Applications? J Urol. (2018) 199:1061–8. doi: 10.1016/j.juro.2017.09.160, PMID: 29174485

[43] Ogunsanya ME Sifat M Bamidele OO Ezenwankwo EF Clifton S Ton. Mobile health (mHealth) interventions in prostate cancer survivorship: a scoping review. J Cancer Surviv. (2023) 17:557–68. doi: 10.1007/s11764-022-01328-3, PMID: 36627464 PMC13014381

[44] Smith AB Samuel CA McCabe SD Deal A Jonsson M Mueller. Feasibility and delivery of patient-reported outcomes in clinical practice among racially diverse bladder and prostate cancer patients. Urol Oncol. (2021) 39:71–7. doi: 10.1016/j.urolonc.2020.06.030, PMID: 32819814 PMC7736202

[45] Dhar E Barsasella D Srikanth S Panja AK Malwade S Syed-Abdul. Using a Wearable Device and Patient Reported Outcome to Evaluate the Influence of Sleep on Quality of Life Among Breast and Prostate Cancer Patients. Stud Health Technol Inform. (2022) 290:526–30. doi: 10.3233/SHTI220132, PMID: 35673071

[46] Wallis CJ Mahar AL Choo R Herschorn S Kodama RT Shah PS. Second malignancies after radiotherapy for prostate cancer: systematic review and meta-analysis. BMJ. (2016) 352:i851. doi: 10.1136/bmj.i851, PMID: 26936410 PMC4775870

[47] Houben LHP Overkamp M Van Kraaij P Trommelen J Van Roermund JGH De Vries P. Resistance Exercise Training Increases Muscle Mass and Strength in Prostate Cancer Patients on Androgen Deprivation Therapy. Med Sci Sports Exerc. (2023) 55:614–24. doi: 10.1249/MSS.0000000000003095, PMID: 36534950 PMC9997646

[48] Victorson D Schalet BD Kundu S Helfand BT Novakovic K Penedo F. Establishing a common metric for self-reported anxiety in patients with prostate cancer: Linking the Memorial Anxiety Scale for Prostate Cancer with PROMIS Anxiety. Cancer. (2019) 125:3249–58. doi: 10.1002/cncr.32189, PMID: 31090933

[49] Lane GI Qi J Dupati A Ferrante S Dunn RL Paudel. Assessing the Impact of Decision Aid Use on Post Prostatectomy Patient Reported Outcomes. Urology. (2022) 165:187–92. doi: 10.1016/j.urology.2022.02.008, PMID: 35219768 PMC9296586

[50] Lane GI Qi J Dupati A Ferrante S Dunn RL Paudel R. Assessing the Impact of Decision Aid Use on Post Prostatectomy Patient Reported Outcomes. Urology. (2022) 165:187–92. doi: 10.1016/j.urology.2022.02.008, PMID: 35219768 PMC9296586

[51] Bandaragoda T Ranasinghe W Adikari A de Silva D Lawrentschuk N Alahakoon D. The Patient-Reported Information Multidimensional Exploration (PRIME) Framework for Investigating Emotions and Other Factors of Prostate Cancer Patients with Low Intermediate Risk Based on Online Cancer Support Group Discussions. Ann Surg Oncol. (2018) 25:1737–45. doi: 10.1245/s10434-018-6372-2, PMID: 29468607

[52] Morgan KM Riviere P Nelson TJ Guram K Deshler LN Sabater Minarim. Androgen Deprivation Therapy and Outcomes After Radiation Therapy in Black Patients With Prostate Cancer. JAMA Netw Open. (2024) 7:e2415911. doi: 10.1001/jamanetworkopen.2024.15911, PMID: 38857047 PMC11165376

[53] Bai J Pugh SL Eldridge R Yeager KA Zhang Q Lee WR. Neighborhood Deprivation and Rurality Associated With Patient-Reported Outcomes and Survival in Men With Prostate Cancer in NRG Oncology RTOG 0415. Int J Radiat Oncol Biol Phys. (2023) 116:39–49. doi: 10.1016/j.ijrobp.2023.01.035, PMID: 36736921 PMC10106367

[54] Jensen RE Potosky AL Moinpour CM Lobo T Cella D Hahn. United States Population-Based Estimates of Patient-Reported Outcomes Measurement Information System Symptom and Functional Status Reference Values for Individuals With Cancer. J Clin Oncol. (2017) 35:1913–20. doi: 10.1200/JCO.2016.71.4410, PMID: 28426375 PMC5466008

[55] van Oosterom MN Meershoek P KleinJan GH Hendricksen K Navab N van de Velde CJH. Navigation of Fluorescence Cameras during Soft Tissue Surgery-Is it Possible to Use a Single Navigation Setup for Various Open and Laparoscopic Urological Surgery Applications? J Urol. (2018) 199:1061–8. doi: 10.1016/j.juro.2017.09.160, PMID: 29174485

[56] Ogunsanya ME Sifat M Bamidele OO Ezenwankwo EF Clifton S Ton C. Mobile health (mHealth) interventions in prostate cancer survivorship: a scoping review. J Cancer Surviv. (2023) 17:557–68. doi: 10.1007/s11764-022-01328-3, PMID: 36627464 PMC13014381

[57] Tran C Dicker A Leiby B Gressen E Williams N Jim H. Utilizing Digital Health to Collect Electronic Patient-Reported Outcomes in Prostate Cancer: Single-Arm Pilot Trial. J Med Internet Res. (2020) 22:e12689. doi: 10.2196/12689, PMID: 32209536 PMC7142743

[58] Dhar E Barsasella D Srikanth S Panja AK Malwade S Syed-Abdul S. Using a Wearable Device and Patient Reported Outcome to Evaluate the Influence of Sleep on Quality of Life Among Breast and Prostate Cancer Patients. Stud Health Technol Inform. (2022) 290:526–30. doi: 10.3233/SHTI220132, PMID: 35673071

[59] George DJ Sartor O Miller K Saad F Tombal B Kalinovský H. Treatment Patterns and Outcomes in Patients With Metastatic Castration-resistant Prostate Cancer in a Real-world Clinical Practice Setting in the United States. Clin Genitourin Cancer. (2020) 18:284–94. doi: 10.1016/j.clgc.2019.12.019, PMID: 32057714

